# Silent but Not Harmless: A Synonymous *SLC5A5* Gene Variant Leading to Dyshormonogenic Congenital Hypothyroidism

**DOI:** 10.3389/fendo.2022.868891

**Published:** 2022-05-04

**Authors:** Romina Celeste Geysels, Carlos Eduardo Bernal Barquero, Mariano Martín, Victoria Peyret, Martina Nocent, Gabriela Sobrero, Liliana Muñoz, Malvina Signorino, Graciela Testa, Ricardo Belisario Castro, Ana María Masini-Repiso, Mirta Beatriz Miras, Juan Pablo Nicola

**Affiliations:** ^1^ Departamento de Bioquímica Clínica, Facultad de Ciencias Químicas, Universidad Nacional de Córdoba, Córdoba, Argentina; ^2^ Centro de Investigaciones en Bioquímica Clínica e Inmunología - Consejo Nacional de Investigaciones Científicas y Técnicas (CIBICI-CONICET), Córdoba, Argentina; ^3^ Programa Provincial de Pesquisa Neonatal, Servicio de Endocrinología, Hospital de Niños de la Santísima Trinidad de Córdoba, Córdoba, Argentina; ^4^ Servicio de Medicina Nuclear, Hospital Nacional de Clínicas, Córdoba, Argentina

**Keywords:** congenital hypothyroidism, iodide transport defect, sodium/iodide symporter, pathogenic synonymous variant, pre-mRNA splicing defect

## Abstract

**Background:**

Congenital iodide transport defect (ITD) is an uncommon cause of dyshormonogenic congenital hypothyroidism characterized by the absence of active iodide accumulation in the thyroid gland. ITD is an autosomal recessive disorder caused by loss-of-function variants in the sodium/iodide symporter (NIS)-coding *SLC5A5* gene.

**Objective:**

We aimed to identify, and if so to functionally characterize, novel ITD-causing *SLC5A5* gene variants in a cohort of five unrelated pediatric patients diagnosed with dyshormonogenic congenital hypothyroidism with minimal to absent ^99m^Tc-pertechnetate accumulation in the thyroid gland.

**Methods:**

The coding region of the *SLC5A5* gene was sequenced using Sanger sequencing. *In silico* analysis and functional *in vitro* characterization of a novel synonymous variant were performed.

**Results:**

Sanger sequencing revealed a novel homozygous synonymous *SLC5A5* gene variant (c.1326A>C in exon 11). *In silico* analysis revealed that the c.1326A>C variant is potentially deleterious for NIS pre-mRNA splicing. The c.1326A>C variant was predicted to lie within a putative exonic splicing enhancer reducing the binding of splicing regulatory trans-acting protein SRSF5. Splicing minigene reporter assay revealed that c.1326A>C causes exon 11 or exon 11 and 12 skipping during NIS pre-mRNA splicing leading to the NIS pathogenic variants p.G415_P443del and p.G415Lfs*32, respectively. Significantly, the frameshift variant p.G415Lfs*32 is predicted to be subjected to degradation by nonsense-mediated decay.

**Conclusions:**

We identified the first exonic synonymous *SLC5A5* gene variant causing aberrant NIS pre-mRNA splicing, thus expanding the mutational landscape of the *SLC5A5* gene leading to dyshormonogenic congenital hypothyroidism.

## Introduction

Congenital iodide transport defect (Online Mendelian Inheritance in Man #274400) is a rare autosomal recessive disorder whose hallmark is the inability of the thyroid follicular cell to actively accumulate the iodide required for thyroid hormonogenesis, thus leading to dyshormonogenic congenital hypothyroidism (1). The general clinical presentation of the disease includes a variable degree of hypothyroidism, reduced to absent radioiodide accumulation in an eutopic thyroid gland, and low saliva-to-plasma iodide ratio (2–4).

The sodium/iodide symporter (NIS) is an integral basolateral plasma membrane glycoprotein that mediates active iodide accumulation into the thyroid follicular cell, which is the first step in the biosynthesis of the iodine-containing thyroid hormones (5). The intracellularly-located carboxy-terminus of the protein is determinant for NIS expression at the basolateral plasma membrane (6, 7). Moreover, NIS-mediated iodide transport is electrogenic (2 sodium:1 iodide stoichiometry) and remarkably efficient considering the submicromolar extracellular iodide concentration (8, 9).

To date, over thirty pathogenic variants in the NIS-coding *SLC5A5* gene have been identified in patients with congenital iodide transport defect. The molecular characterization of several disease-causing NIS variants has revealed critical amino acids for substrate binding, specificity, and stoichiometry, as well as folding and plasma membrane targeting (5). A detailed analysis of the p.G561E NIS variant revealed the importance of the kinesin-1 subunit kinesin light chain 2 (KLC2) in thyroid hormonogenesis (10). Moreover, the functional characterization of the p.D396V NIS variant uncovered a critical intramolecular ionic interaction—involving the β carboxyl group of D369 and the guanidinium group of R130—for the correct folding required for NIS maturation and transport to the plasma membrane (11). Recently, based on structure-function analysis of pathogenic NIS variants, we developed a machine learning-based NIS-specific variant classifier aiming to improve the prediction of pathogenicity of missense NIS variants in clinical practice (12).

Here, we explored the presence of pathogenic *SLC5A5* gene variants in a cohort of five unrelated pediatric patients with dyshormonogenic congenital hypothyroidism suspected of having a congenital iodide transport defect based on reduced to non-detected ^99m^Tc-pertechnetate accumulation in an eutopic thyroid gland. We identified the homozygous synonymous *SLC5A5* variant c.1326A>C. *In silico* analysis predicted that c.1326A>C disrupts the binding of the splicing regulatory protein SRSF5 to an exonic splicing enhancer located in exon 11. Functional *in vitro* characterization using a splicing minigene reporter assay revealed that the c.1326A>C variant causes exon 11 or exons 11 and 12 skipping during NIS pre-mRNA splicing leading to the NIS pathogenic variants p.G415_P443del and p.G415Lfs*32, respectively. Significantly, the frameshift variant p.G415Lfs*32 is predicted to be subjected to degradation by nonsense-mediated decay.

## Material and Methods

### Patients

Five patients with dyshormonogenic congenital hypothyroidism with a suspected defect in iodide accumulation were included in the study. All patients were full-term infants born from non-consanguineous Caucasian (of European descent) parents showing an abnormally high TSH level during the neonatal screening program (**Table 1**). The patients did not have clinical signs of hypothyroidism at birth. The cut-off value adopted for basal TSH level in blood spot determined by UMELISA TSH Neonatal (TecnoSuma International SA, La Habana, Cuba) for neonatal screening was 10 µIU/ml. The diagnosis of congenital hypothyroidism was confirmed based on elevated TSH serum levels, with or without total or free T4 levels below the normal range (**Table 1**). Inclusion criteria were congenital hypothyroidism with eutopic thyroid gland explored by ultrasonography and reduced to absent ^99m^Tc-pertechnetate accumulation in the thyroid gland assessed by radionuclide scintigraphy. The reference range for ^99m^Tc-pertechnetate uptake adopted in the Division of Nuclear Medicine is 0.5–4% (13); therefore, we considered values lower than 0.4% to be reduced, and suggestive of defective iodide accumulation. The accumulation of ^99m^Tc-pertechnetate in salivary glands was not tested. Determination of saliva-to-plasma iodide ratio was not technically available in the Division of Nuclear Medicine. Levothyroxine therapy was introduced immediately after diagnosis at initial dosis of 5–15 µg/kg per day according to the degree of hypothyroidism. Patient 2 was re-evaluated at the age of three years as recommended by consensus guidelines (14); discontinuation of levothyroxine treatment revealed transient hypothyroidism.

**Table 1:** Summary of biochemical and imaginological findings.

Patient	1	2	3	4	5
**Neonatal screening**					
Age (days)	36	7	4	7	4
TSH (<10 mU/l)	>20	18	20	29	67
**Biochemical analysis**					
Age (days)	49	25	15	50	15
TSH (0.8-7.8 μg/dl)	300	16	16	46	62
T_4_ (6-16.5 μg/dl)	2	5	8	9	4
Free T_4_ (1-2.1 ng/dl)	0.1	1.3	1.2	1.0	1.2
T_3_ (100-310 ng/dl)	45	156	155	218	164
Tg (6-83 ng/ml)	5.2	59	221	67	157
Anti-TPO/Tg antibodies	Negative	Negative	Negative	Negative	Negative
**Imaginological analysis**					
Ultrasonography	Eutopic	Eutopic	Eutopic	Eutopic	Eutopic
^99m^Tc-pertechnetate scintigraphy	Negative*	Negative	Reduced	Reduced	Reduced

* ^99m^Tc-pertechnetate scintigraphy was performed 8 days after initiation of levothyroxine replacement therapy.

### Thyroid Function Tests

Thyroid function analyses were performed by electrochemiluminescence immunoassay by Cobas e412 analyzer (Roche Diagnostics, Indianapolis, IN). Age-specific thyroglobulin reference intervals were established in-house (15).

### *SLC5A5* Gene Sequencing

Genomic DNA was extracted from whole blood using the standard cetyltrimethylammonium bromide-based method. All 15 coding exons and exon-intron boundaries of the *SLC5A5* gene were amplified using the primers and PCR conditions previously reported (16, 17). The nucleotide sequence of all PCR products was determined in both orientations by Sanger sequencing by capillary electrophoresis (Macrogen, Seoul, South Korea). The nucleotide position in *SLC5A5* mRNA was named according to the National Center for Biotechnology Information reference sequence NM_000453.3, considering the adenine of the ATG translation start codon as nucleotide +1.

### *In Silico* Analysis

Splice Site Prediction by Neural Network (NNSplice), Max Entropy Scan (MES), GeneSplicer, Human Splicing Finder (HSF), SpliceSiteFinder-like (SSF-like), ESEFinder and RESCUE-ESE integrated into the splicing module of Alamut Visual version 2.9.0 (Interactive Biosoftware, Rouen, France) were used to investigate the effect of synonymous variants on consensus acceptor and donor splice-site sequences at the intron-exon boundaries and exonic splicing regulatory elements. Additional data analysis to assess the effect of synonymous variants on exonic splicing regulatory elements was accomplished using the algorithm HExoSplice (18). The algorithm NMDEscPredictor (19) was used to predict whether frameshift variants are sensitive to nonsense-mediated mRNA decay.

### Cloning and Site-Directed Mutagenesis

Human genomic sequence containing exons 11 (87 nucleotides) and 12 (197 nucleotides) of the *SLC5A5* gene along with the last 311 nucleotides of intron 10, the 82 nucleotides of intron 11, and the first 367 nucleotides of intron 12 were amplified by PCR using the following primers containing XhoI and BamHI restriction sites (underlined) 5’- CACACTCGAGGTTGCAGTGAGCCAAGATCG (forward) and 5’- TGTGGGATCCTCAAGCTGGGAGGATTGC (reverse). PCR primers were designed using the Primer3 server (http://frodo.wi.mit.edu/). The DNA fragments were cloned into the corresponding cloning sites of the splicing reporter pSPL3 vector (a discontinued product of Thermo-Fisher Scientific) (20). Site-directed mutagenesis was performed by PCR with oligonucleotides carrying the desired mutation using Phusion Hot Start II DNA Polymerase (Thermo-Fisher Scientific), which was followed by template plasmid digestion with DpnI (Promega – Madison, WI) (21). Oligonucleotides for site-directed mutagenesis were designed using QuikChange Primer Design Program (Agilent Technologies, Santa Clara, CA). All constructs were sequenced to verify specific nucleotide substitutions (Macrogen).

### Cell Culture and Transfections

HeLa cells (CCL-2, American Type Culture Collection, Rockville, MD) were obtained from our institutional cell line repository. Cells were cultured in Dulbecco Modified Eagle’s Medium (Thermo-Fisher Scientific - Waltham, MA) supplemented with 10% fetal bovine serum (Natocor, Córdoba, Argentina). Cells were transiently transfected at the ratio of 4 µg plasmid/10 cm dish using Lipofectamine 2000 (Thermo-Fisher Scientific) (22).

### Splicing Minigene Reporter Assays

Total RNA was extracted from Hela cells 24 hours after transient transfection with pSPL3-based minigene reporters using the Direct-zol RNA MiniPrep Kit (Zymo Research - Irvine, CA). Complementary DNA was synthesized from 1 µg total RNA. PCR reactions were performed as described (23). The pSPL3-specific primer sets were as follows: SD6 5′- TCTGAGTCACCTGGACAACC and SA2 5′- ATCTCAGTGGTATTTGTGAGC. RT-PCR products were resolved by electrophoresis on 2.5% agarose gels containing ethidium bromide and gel purified using Wizard SV Gel and PCR Clean-Up System (Promega) according to the manufacturer’s protocol. All PCR products were sequenced to verify the identity of spliced exons (Macrogen).

## Results

### Identification of Synonymous *SLC5A5* Variants in Patients With Dishormonogenic Congenital Hypothyroidism

Five patients with congenital hypothyroidism showing diffuse and reduced to non-detectable ^99m^Tc-pertechnetate uptake by the thyroid gland, which suggested a congenital iodide transport defect, were included in the study (**Table 1**). The presence of an eutopic normal-shaped thyroid gland was ascertained by ultrasonography. All patients were detected through neonatal screening assessing TSH levels (**Table 1**). Confirmatory thyroid function test showed increased TSH and normal to reduced total and free thyroxine (T4) serum levels. Thyroglobulin serum levels were variable and all patients were tested negative for thyroid autoantibodies (**Table 1**).

**Figure 1:**
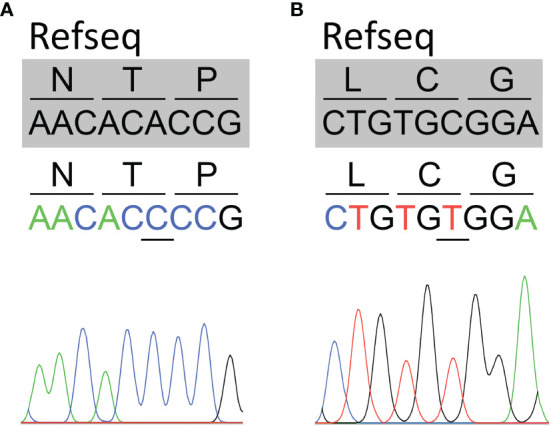
Identification of synonymous *SLC5A5* variants in patients with dyshormonogenic congenital hypothyroidism. Partial sequence chromatograms covering the region of the variants (underlined) in exons 11 (c.1326A>C) **(A)** and 13 (c.1626C>T) **(B)** of the *SLC5A5* gene. The reference sequence (Refseq) is indicated in gray.

Patients’ *SLC5A5* gene sequencing revealed the homozygous synonymous variants c.1326A>C located in exon 11 (patient 4) (**Figure 1A**) and c.1626C>T located in exon 13 (patient 2) (**Figure 1B**), whereas no variants were identified in the remaining patients. The variants c.1326A>C and c.1626C>T have been reported in the Single Nucleotide Polymorphism database (rs73520743 and rs45602038, respectively). The variant c.1326A>C showed an allele frequency of 0.001103 in the European (non-Finnish) population, while c.1626C>T was 0.02997 in the same population, according to The Genome Aggregation Database (accessed on August 2021). We did not observe the variants in the genome of 50 age-matched healthy pediatric patients. Moreover, the variants c.1326A>C and c.1626C>T were observed in homozygosis in 1 and 56, respectively, out of 61659 European (non-Finnish) individuals, according to The Genome Aggregation Database (accessed on August 2021). Hence, according to the standards and guidelines provided by the American College of Medical Genetics and Genomics (24), the c.1626C>T variant was classified as benign (BS1).

### *In Silico* Analysis Predicts the Synonymous c.1326A>C Variant as Deleterious for Normal NIS Pre-mRNA Splicing

Synonymous variants are frequently considered benign as these do not alter the corresponding amino acid residue and have no direct effect on the protein sequence. However, synonymous variants may impact on transcription, mRNA processing and translation (25).

*In silico* analysis using splicing prediction algorithms revealed that the c.1326A>C variant, located at position +4 of the exon/intron 11 junction, has no impact on the splice consensus sequence nor the creation of a new splice site. However, complementary *in silico* analysis assessing the effect of the c.1326A>C variant on splicing regulatory elements revealed that the variant is potentially deleterious for normal NIS pre-mRNA splicing. The variant c.1326A>C was predicted to lie at a potential exonic splicing enhancer (ESE) motif (ACAC[A/C]CC) using ESEfinder software. The motif carrying the c.1326A>C variant exhibited a decrease in the splicing regulatory protein SRSF5 binding score from 4.12 to below the threshold level set at 2.67. In addition, the analysis using HExoSplice software revealed that the variant c.1326A>C causes a significant negative effect on an exonic splicing regulatory (ESR) sequence (ΔtESRseq = -0.5932) whose identity overlaps with the ESEfinder-predicted SRSF5 binding site. Together, *in silico* analysis suggests that the synonymous variant c.1326A>C might reduce the affinity of SRSF5 binding to the mutant ESE motif. In agreement, previous reports suggested that variants in ESE could cause exon skipping leading to aberrant pre-mRNA splicing (26–28).

### The Pathogenic Variant c.1326A>C Impairs Normal NIS Pre-Messenger RNA Splicing

**Figure 2:**
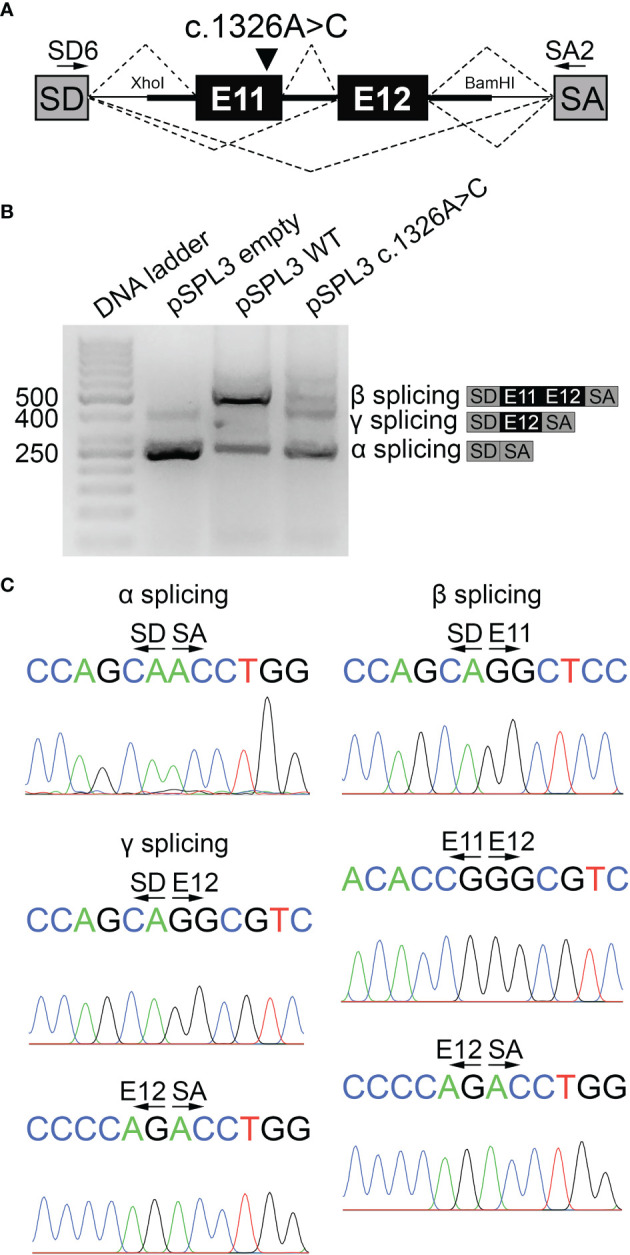
The variant c.1326A>C causes NIS pre-mRNA splicing defect. **(A)** Scheme of pSPL3-based minigene reporter constructs used in functional assays. The genomic fragment containing exons 11 and 12 along with a fragment flanking introns 10 and 12, and the spacing intron 11 was cloned between pSPL3 vector exons SD and SA using XhoI and BamHI restriction sites. Arrows show pSPL3 vector SD and SA exon-specific primers (SD6 and SA2) used in RT-PCR analysis. Canonical and aberrant splicing products are indicated by dashed lines above and below the construct, respectively. The variant c.1326A>C is indicated. **(B)** Agarose gel electrophoresis of RT-PCR products from empty, WT or c.1326A>C pSPL3 minigene reporter transiently transfected into HeLa cells. The empty pSPL3 vector, where only SD-SA exons splicing occurred, lead to a 263 bp PCR product (α splicing). The WT pSPL3 minigene mainly yielded a 547 bp PCR product including SD-SA exons (263 bp) flanking the exons 11 (87 bp) and 12 (197 bp) of the *SLC5A5* gene (β splicing). The c.1326A>C pSPL3 minigene leads to a main PCR product of 263 bp including only SD-SA exons and secondary PCR products of 457 and 573 bp including SD-SA exons flanking exon 12 alone (γ splicing) or including an artificial pseudoexon derived from the intronic sequence of the reporter vector (**Supplementary Figure 1**), respectively. Schemes represent the α, β and γ splicing RT-PCR products. **(C)** Sequencing analysis confirmed the identity of α, β and γ splicing RT-PCR products.

To functionally assess the impact of the synonymous c.1326A>C variant, pSPL3-based minigene reporter constructs were generated and functionally tested in transiently transfected HeLa cells (**Figure 2A**). Minigene reporter assay revealed that the variant c.1326A>C generates a transcript of 263 bp compatible in size with the skipping of exons 11 and 12 (α splicing product), as a similar pattern was observed when cells were transfected with the empty reporter vector (**Figure 2B**). By contrast, the WT minigene generates a transcript of 547 bp compatible with the canonical spliced transcript including exons 11 and 12 (β splicing product) and a transcript of 263 bp (α splicing product) (**Figure 2B**). Moreover, the variant c.1326A>C also generated a transcript of 457 bp compatible with the spliced transcripts including exon 12 alone (γ splicing product) (**Figure 2B**), and an additional artificial splicing transcript of 573 bp including a pseudoexon from the intron of the minigen reporter vector (**Supplementary Figure 1**). Sequence analysis uncovered the identity of all PCR products (**Figure 2C** and **Supplementary Figure 1**).

If the exon-excluded transcripts of the *SLC5A5* gene were successfully translated, as the c.1326A>C variant-caused exons 11 and 12 skipping induces a change in the open reading frame of the transcript, it would lead to the NIS variant p.G415Lfs*32, whereas exon 11 skipping would generate the in-frame NIS deletion variant p.G415_P443del. Significantly, NMDEscPredictor analysis predicted that the transcript encoding the frameshift variant p.G415Lfs*32 undergoes nonsense-mediated decay.

Together, combining *in silico* and functional *in vitro* assays, we determined that the synonymous c.1326A>C variant disturbed normal NIS pre-mRNA splicing, thus leading to dyshormonogenic congenital hypothyroidism due to a defect in iodide accumulation.

## Discussion

Here, we explored the presence of pathogenic *SLC5A5* gene variants in a cohort of five unrelated pediatric patients with dyshormonogenic congenital hypothyroidism suspected of having a congenital iodide transport defect. Significantly, we identified the homozygous synonymous c.1326A>C variant causing aberrant NIS pre-mRNA splicing in one of the patient. Consistent with the recessive nature of the disease, the mother of the patient showed conserved thyoid function although her genetic material was not available for segregation analysis. Our analysis did not reveal pathogenic variants in the coding region of the *SLC5A5* gene in three out of four patients with permanent congenital hypothyroidism. Therefore, although we focused on the coding region and exon-intron boundaries, other potential mechanisms, such as large genomic rearrangements and variants likely affecting regulatory elements, including the promoter, enhancers, and deep intronic regions should also be considered. Interestingly, pathogenic variants in *TSHR* and *PAX8* genes have been reported in patients with permanent congenital hypothyroidism showing reduced to normal-sized eutopic thyroid glands with low to absent radioiodide uptake on thyroid scintigraphy (29, 30). Regarding the patient with transient congenital hypothyroidism, Castellnou et al. (31) recently reported a case of transient congenital hypothyroidism with eutopic gland and undetectable radioiodide uptake on thyroid scintigraphy due to maternal thyrotropin receptor-blocking antibodies (TRAbs). This report highlights the importance to analyze TRAbs in hypothyroid mothers during pregnancy, which is particularly useful when congenital hypothyroidism is diagnosed in their newborns. Unfortunately, maternal TRAbs data were not available for our patients.

Most disease-causing exonic single nucleotide variants are frequently assumed to exert their effects by altering the amino acid sequence of the protein. However, many human genetic diseases are caused by exonic variants that disrupt canonical splice sites at exon-intron boundaries or splicing regulatory elements that enhance the recognition of the splice sites, which are relevant to pre-mRNA splicing, leading to abnormal splicing outcomes (32). Indeed, one-third of disease-causing mutations were predicted to result in aberrant splicing (33). Interestingly, exonic synonymous variants—which do not alter protein sequences—or even non-synonymous single nucleotide variants have been demonstrated to contribute to human diseases by affecting transcription and splicing regulatory factors within protein-coding regions (33). Particularly, synonymous variants were reported as a disease-causing mechanism in several endocrine disorders including familial pheochromocytoma (34), combined pituitary hormone deficiency (35, 36), pseudohypoparathyroidism type 1 (37) and maturity-onset diabetes of the young (38). Therefore, synonymous variants should not be neglected in gene variant prioritization pipelines as they may produce abnormal mRNAs and dysfunctional proteins.

Patients with congenital hypothyroidism due to deficient iodide accumulation show a substantial clinical and biochemical heterogeneity (39). Under conditions of iodine sufficiency, thyroid function at diagnosis reflects differences in residual mutant NIS protein activity (39, 40). Thus, thyroid function in the patient carrying the pathogenic variant c.1326A>C was sufficient to preserve normal peripheral thyroid hormone levels. A likely explanation for this situation is the presence of residual properly-spliced NIS expression, which correlates with the clinical observation of reduced ^99m^Tc-pertechnetate when the patient was examined by thyroid scintigraphy. Considering that NIS-mediated iodide uptake is a crucial step in the synthesis of thyroid hormones, our results indicate that the variant c.1326A>C impairs iodide uptake by interfering with normal NIS pre-mRNA splicing. Therefore, defective NIS pre-mRNA splicing leading to lack of sufficient NIS molecules at the basolateral plasma membrane in the thyroid follicular cells may explain the mechanism underlying the deficient accumulation of iodide causing dyshormonogenic hypothyroidism. In this context, the increase in TSH levels following a decrease in thyroid hormone production may partially overcome the splicing defect by upregulating *SLC5A5* gene transcription, as has been observed in patients with different loss-of-function NIS variants (41, 42).

Disruption of pre-mRNA splicing has been implicated in the etiology of numerous congenital human disorders (43). Several reports support splicing defects as a disease-causing mechanism in congenital hypothyroidism (44–48). Particularly, NIS splicing defect-caused dyshormonogenic congenital hypothyroidism has been described in the literature. Pohlenz et al. (49) reported the nonsense variant c.1593C>G (originally named c.1940C>G) that generates a cryptic 3’ splice acceptor site in exon 13 leading to the mis-splicing variant p.S509Rfs*6 NIS. Moreover, two recent reports provided functional evidence that the variants c.970-3C>A and c.970-48G>C disrupts canonical and non-canonical splice sites, respectively, located in the intron 7 causing exon 8 skipping during NIS mRNA splicing, thus leading to the nonsense variant p.Y324Hfs*148 NIS (11, 50). Here, we report the exonic synonymous *SLC5A5* gene variant c.1326A>C causing aberrant NIS pre-mRNA splicing. The variant c.1326A>C is predicted to disrupt a potential ESE motif located in exon 11, thus promoting exon 11 or exons 11 and 12 skipping during the splicing process. The skipping of exon 11 during NIS pre-mRNA splicing leads to the in-frame deletion variant p.G415_P443del, which is likely inactive, as the ITD-causing in-frame deletion variant p.A439_P443del NIS identified in a patient showing not-detectable ^131^I-iodide uptake by the thyroid gland is intracellularly retained and does not accumulate iodide (51, 52). Moreover, the skipping of exons 11 and 12 change the open reading frame of the transcript leading to the NIS pathogenic variant p.G415Lfs*32, whose transcript is predicted to undergo nonsense-mediated mRNA decay.

The implementation of next-generation sequencing has been instrumental in expanding the mutational landscape of monogenic forms of congenital hypothyroidism (53). Recently, whole-exome sequencing revealed pathogenic *GBP1* variants in patients with defective developmental thyroid morphogenesis (54). The future implementation of next-generation sequencing-based approaches might reveal novel disease-causing gene variants in patients with permanent dyshormonogenic congenital hypothyroidism showing a phenotype of defective iodide accumulation. A precedent for this hypothesis is provided by the observation that the KCNQ1/KCNE2 potassium channel is crucial in facilitating NIS-mediated iodide transport (55).

## Data Availability Statement

The raw data supporting the conclusions of this article will be made available by the authors, without undue reservation.

## Ethics Statement

The studies involving human participants were reviewed and approved by the medical ethics committee of the Hospital de Niños de la Santísima Trinidad (Córdoba, Argentina). Written informed consent to participate in this study was provided by the participants’ legal guardian/next of kin.

## Author Contributions

RG performed research and analyzed the data. CBB, MM, VP, MN, and RC provided critical technical support and analyzed the data. GS, LM, MS, GT, and MM diagnosed and followed-up the patients enrolled in the study. AM-R, MM, and JN conceived the project. RG and JN designed research. JN drafted the manuscript. All authors edited and approved the final version of the manuscript.

## Funding

This work was supported by Fondo para la Investigación Científica y Tecnológica - Agencia Nacional de Promoción Científica y Tecnológica (grants number PICT-2018-1596 and PICT-2019-1772), Secretaría de Políticas Universitarias - Ministerio de Educación (grant number VT12-UNCOR4153), and Secretaría de Ciencia y Tecnología - Universidad Nacional de Córdoba (grant number 33620180100772CB).

## Conflict of Interest

The authors declare that the research was conducted in the absence of any commercial or financial relationships that could be construed as a potential conflict of interest.

## Publisher’s Note

All claims expressed in this article are solely those of the authors and do not necessarily represent those of their affiliated organizations, or those of the publisher, the editors and the reviewers. Any product that may be evaluated in this article, or claim that may be made by its manufacturer, is not guaranteed or endorsed by the publisher.
