# Supplementary material for: Silent but Not Harmless: A Synonymous SLC5A5 Gene Variant Leading to Dyshormonogenic Congenital Hypothyroidism
Source: Front Endocrinol (Lausanne). 2022 May 4;13:868891. doi: 10.3389/fendo.2022.868891 (PMC9114739; doi:10.3389/fendo.2022.868891)
Supplement: Supplementary file 1 [file DataSheet_1.pdf]

![frontiers logo]

Supplementary Material

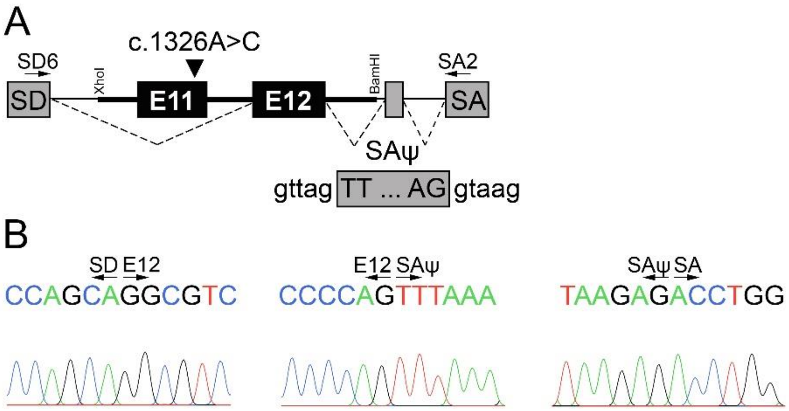

Supplementary Figure 1. Artificial pseudoexon inclusion. (A) Scheme of pSPL3-based minigene reporter constructs used in functional assays. The pSPL3 vector contains a small artificial gene composed of an exon-intron-exon sequence with functional splice donor and acceptor sites. The single intron harbors a multiple cloning site where the genomic fragment of interest containing exons 11 and 12 along with a fragment of flanking introns 10 and 12, and the spacing intron 11 was cloned using XhoI and BamHI restriction sites. The variant c.1326A>C and pSPL3 vector SD and SA exon-specific primers (SD6 and SA2) used in RT-PCR analysis are indicated. The artificial splicing RT-PCR transcript of 573 bp containing a 116 bp sequence derived from the inclusion of a pseudoexon (SAψ) within the intron downstream the cloning site is indicated by dashed lines below the construct. We use the term pseudoexon to indicate an artificial exon, containing adequate 5′ and 3′ splice sites, as determined by the splice site prediction algorithm Spliceator (www.lbgi.fr/spliceator/). **(B)** Sequencing analysis confirmed the identity of the artificial RT-PCR product including the pseudoexon SAψ.
